# *Bicaudal C* mutation causes *myc* and TOR pathway up-regulation and polycystic kidney disease-like phenotypes in *Drosophila*

**DOI:** 10.1371/journal.pgen.1006694

**Published:** 2017-04-13

**Authors:** Chiara Gamberi, David R. Hipfner, Marie Trudel, William D. Lubell

**Affiliations:** 1 Department of Biology, Concordia University, Montréal, QC, Canada; 2 Institut de recherches cliniques de Montréal, 110 Pine Avenue West, Montréal, QC, Canada; 3 Département de médecine, Université de Montréal, Montréal, QC, Canada; 4 Département de chimie, Université de Montréal, Montréal, QC, Canada; New York University, UNITED STATES

## Abstract

Progressive cystic kidney degeneration underlies diverse renal diseases, including the most common cause of kidney failure, autosomal dominant Polycystic Kidney Disease (PKD). Genetic analyses of patients and animal models have identified several key drivers of this disease. The precise molecular and cellular changes underlying cystogenesis remain, however, elusive. *Drosophila* mutants lacking the translational regulator Bicaudal C (BicC, the fly ortholog of vertebrate BICC1 implicated in renal cystogenesis) exhibited progressive cystic degeneration of the renal tubules (so called “Malpighian” tubules) and reduced renal function. The BicC protein was shown to bind to *Drosophila* (*d-) myc* mRNA in tubules. Elevation of d-Myc protein levels was a cause of tubular degeneration in *BicC* mutants. Activation of the Target of Rapamycin (TOR) kinase pathway, another common feature of PKD, was found in *BicC* mutant flies. Rapamycin administration substantially reduced the cystic phenotype in flies. We present new mechanistic insight on *BicC* function and propose that *Drosophila* may serve as a genetically tractable model for dissecting the evolutionarily-conserved molecular mechanisms of renal cystogenesis.

## Author summary

Polycystic kidney disease (PKD) is a degenerative, potentially lethal, genetic malady that affects 12.5 million people world-wide for which there is no cure. In the kidney, PKD causes the formation of prominent, fluid-filled cysts the growth of which damages progressively kidney function. Crucial to PKD development, mutations in the *PKD1* and *PKD2* genes cause renal cystic degeneration via factors and mechanisms that are only partially known. This manuscript reports novel insights into the molecular mechanisms of the evolutionarily conserved RNA binding protein BicC, which has been implicated in vertebrate cystic kidney diseases. The *BicC* mutants of the fruit fly *Drosophila melanogaster* recapitulate crucial characteristics of PKD. A clear link between *BicC* and PKD has begun to emerge, in part because both *PKD1* patients and *Pkd1* mice exhibit reduced *BicC* function. This first in kind *Drosophila* model of renal cystogenesis offers strong potential to decipher the complex mechanisms of the molecular and cellular changes causing renal cyst formation.

## Introduction

Maintenance of structural and functional integrity of the kidney is a complex, crucial task presided over by the activity of numerous genes. Renal cyst formation can result from the mutation of at least one of over 57 genes [1,2]. Multiple, clinically relevant, forms of cystic kidney disease exist, exhibiting different modalities of genetic inheritance: syndromic, non-syndromic, dominant, and recessive [2]. Autosomal Dominant Polycystic Kidney Disease (ADPKD) is the most common cause of end-stage renal failure, affects 12.5 million people world-wide and has an incidence of 1–2 cases per 2000 live births world-wide [1]. Mutations in the *PKD1* and *PKD2* genes account for the majority of the genetic lesions in ADPKD patients [3,4]. ADPKD causes the loss of polarity in the cells of the renal tubule epithelium and the development of fluid-filled cysts and interstitial fibrosis in the kidney [1,5,6,7]. Several animal models have been studied to identify the pathways and the complex changes that eventually lead to renal cystogenesis [8]. Among these, activation of the mammalian (m) TOR pathway was found in various forms of renal cystic pathologies, including human ADPKD cysts, autosomal recessive PKD, and rodent models of PKD and nephronophthisis [5,6,7,9,10,11]. TOR is a conserved serine threonine kinase central to controlling cellular anabolic processes via protein translation, ribosome biogenesis, nutrient sensing/transport and mitochondrial metabolism [12,13,14]. TOR is often dys-regulated in disease [12,15]. The stages and pathological progression of PKD cystogenesis are known [1]. Although genetic analyses have indicated that some signaling pathways are altered in PKD tissue, the precise molecular and cellular changes underlying cystogenesis in PKD and other diseases remain elusive.

Mutations in the *Bicaudal C* (*BicC*) gene in many vertebrates are associated with the development of renal cysts [16,17,18,19]. BicC proteins are a family of RNA binding factors of which the prototypical member, BicC, was demonstrated to be necessary for establishing the correct anterior-posterior polarity of the embryo of the fruit fly *Drosophila melanogaster* [20,21]. Subsequent studies reported that BicC functions to establish polarity of the oocyte, and consequently of the embryo. During oogenesis BicC recruits the CCR4-NOT deadenylase to its target mRNAs and regulates cytoplasmic polyadenylation, eventually affecting translation [22,23,24].

Evolutionarily conserved from *Drosophila* to vertebrates, *BicC* is abbreviated as *Bicc1* and *BICC1* for mouse and humans respectively [20]. Several lines of evidence have implicated Bicc1 and BICC1 in renal function and cystogenesis. Mutations in *Bicc1* as well as in its *Xenopus* and zebrafish orthologs result in cystic kidneys [16,19,25,26]. In humans, two *BICC1* mutations were identified in patients with cystic kidney dysplasia [17]. Bicc1 can associate with the Ank and NPHP proteins in a complex important for the development of the zebrafish pronephros [27]. The Bicc1/Ank/NPHP complex was implicated in human nephronophtisis, a cystic kidney disease characterized by multiple extra-renal manifestations [28]. Moreover, a 30% reduction in *Pkd2* mRNA levels has been observed in *Bicc1* mutant mice [25]. Despite the numerous vertebrate *BicC* animal models, the precise molecular mechanisms underlying BicC-dependent kidney degeneration are largely unknown.

The *Drosophila* renal function is carried out by two pairs of renal tubules, specifically called Malpighian tubules [29], and anatomically separated nephrocytes, that resemble vertebrate podocytes [30]. The fly Malpighian tubules are regarded as morphologically and functionally equivalent to the vertebrate renal tubule. Fly and vertebrate tubules both are composed of a tubular epithelium, contain distinct physiological regions, produce primary urine and reabsorb some solutes [31,32]. The transcriptomes of the Malpighian tubule and the human renal tubule are also remarkably similar [33]. Several fly models exist for various kidney diseases [34] because of the evolutionary conservation of renal function; however, no *Drosophila* models have been described for cystic kidney disease.

Herein, we report that *Drosophila BicC* mutants developed cysts in the Malpighian tubules and provide novel evidence of the mechanism of BicC regulation. BicC was found to bind directly the *d-myc* mRNA. Characteristic of cystic tubule degeneration and PKD, *BicC* mutants exhibited both *d-myc* up-regulation and TOR pathway activation. Moreover, underscoring conserved causative mechanisms, rapamycin, a TOR inhibiting drug known to ameliorate PKD defects in vertebrates, was shown to be effective in reducing the defects observed in *BicC* mutant flies. Notably, *BicC* was significantly down-regulated in PKD tissue, both in patients and in mouse *Pkd1* models, indicating that *BicC* is genetically downstream of *PKD1*. Therefore, *Drosophila BicC* mutants appear to provide a valid model to dissect the molecular and cellular aspects of cyst biology.

## Results

### Impaired *BicC* function induces renal cystogenesis in *Drosophila*

**Fig 1:**
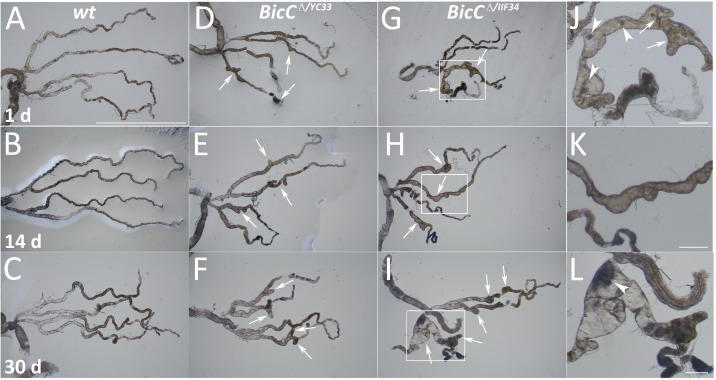
*BicC* is necessary for proper renal tubule function. Malpighian (fly renal) tubules were dissected and photographed (light microscopy) at 1 day (A, D, G) or 14 days (B, E, H) or 30 days (C, F, I). Compared to *Ore*^*R*^ wild-type flies (*wt*, A-C), the *BicC*^*Δ/YC33*^ (D-F) and the *BicC*^*Δ/IIF34*^ (G-I) mutant flies both displayed cystic tubules. Both Malpighian tubules degenerated over time, with cysts evident throughout the whole tubule and in both tubules by day 14 (compare A, D, G with B, E, H and C, F, I). Enlargements of the boxed areas in (G, H, I) are shown respectively in (J, K, L), highlighting the tubular deformities. The normal single-cell layer of the renal tubule epithelium appeared disorganized in the mutant flies, which had abnormal tubule budding and branching (arrows). Malpighian tubules dissected *ex vivo* from *BicC* mutants contained various crystalline particles apparently floating in the luminal fluid (arrowheads) that were not observed in wild-type flies raised in parallel under identical conditions. Scale bar: 1 mm (A-I) or 312.5 μm (J, K, L).

As seen by light microscopy, *wild-type* Malpighian tubules were thin and elongated, with regular diameter. This appearance was constant over thirty days of adult life (Fig 1A–1C). In contrast, *BicC* mutant tubules from *Df(2L)RA5/BicC*^*YC33*^ and *Df(2L)RA5/BicC*^*IIF34*^ flies (subsequently referred to as *BicC*^*Δ/YC33*^ and *BicC*^*Δ/IIF34*^ respectively) exhibited deformations from early age (1 day, Fig 1D–1F and 1G–1I, respectively). Disorganization of the tubular epithelial cells and irregularly distributed enlargements were consistently observed in the *BicC* mutant flies.

**Fig 2:**
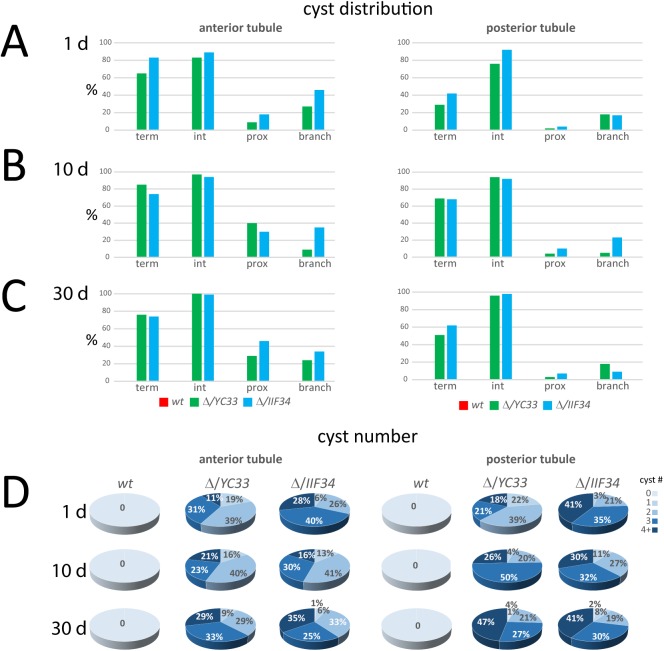
Quantification of the Malpighian tubule cystic phenotype of *BicC* flies. Malpighian tubules from wild type, *BicC*^Δ/*YC33*^ and *BicC*^Δ/*IIF34*^ (50 flies each) were dissected at age one day (A), 10 days (B), and 30 days (C) and scored phenotypically *ex vivo*. Shown are the percentages of tubules affected in the terminal, intermediate, and proximal regions, as well as the observed extra tubular branching. Anterior and posterior tubules were scored separately. Tubular cyst number from the same sample is shown (D). The occurrence of *BicC*^*Δ/YC33*^ tubules displaying at least four cysts appeared to increase over time. The same class in the *BicC*^*Δ/IIF34*^ tubules was more numerous at one day of age, was less represented at ten days, and increased again in the older flies (Fig 2D), possibly reflecting mortality of the more severely affected flies (see text).

The Malpighian tubule malformations were reminiscent of the defects observed in various forms of human PKD [35]. At an early age the tubules appeared severely affected and presented large, often numerous cysts in both *BicC*^*Δ/YC33*^ and *BicC*^*Δ/IIF34*^ flies (Fig 1D, 1G and 1J). When the *BicC* mutant flies approached 14 days of age, both tubules displayed irregularities, appeared enlarged, and presented numerous deformities (Fig 1E, 1H and 1K). Prominent enlargements closely resembling cysts appeared filled with fluid and floating dark crystalline particles that were not observed in the wild-type controls that were raised in parallel under identical conditions. Dense, amorphous, material could also be seen in long sections of the *BicC* tubules and -rarely- in short sections of the wild-type tubules from old flies. Unlike wild type, that maintained thin, elongated Malpighian tubules, at 30 days, *BicC* flies displayed multiple cysts in both tubules and prominent deformities of the anterior terminal tubule (Fig 1C, 1F, 1I and 1L). Additionally, extra tubular budding and branching were observed exclusively in *BicC* mutant tubules (Fig 1J and 1L). To better characterize the defects and the progression of the phenotype over time, we scored the incidence of cystic deformations in Malpighian tubules dissected from 50 flies (100 anterior and 100 posterior tubules) of each genotype and at different ages. Both pairs of Malpighian tubules displayed cysts, especially in the terminal and intermediate regions (observed in 42–100% of cases, Fig 2A–2C), which was reminiscent of PKD that is reported to affect preferentially the terminal section of the nephron [36,37]. Akin to PKD [36] the *BicC* tubules also presented extra branching (9–46% of cases). The cysts of the *BicC* Malpighian tubules appeared to become more numerous over time, albeit the deaths of many flies influenced the representation of the different classes (Fig 2D).

**Fig 3:**
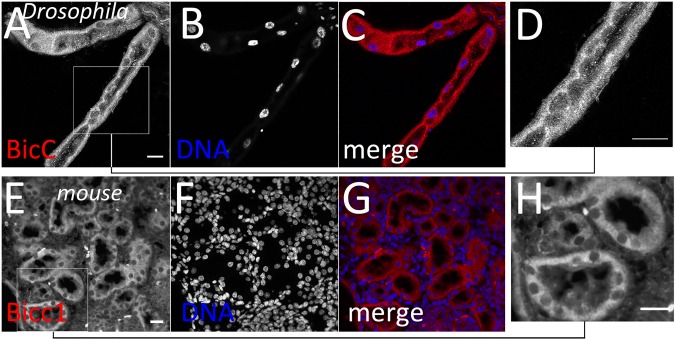
BicC expression in renal tissue. Confocal section of *Drosophila* Malpighian tubules with BicC immunofluorescent staining (red, A, C, D) and DAPI nuclear staining (blue B, C) show that BicC is expressed in the principal cells. Boxed area in (A) is shown enlarged in (D). Epifluorescence microscopy of a 5 μm kidney section from C57BL/6 mice shows Bicc1 accumulation in the cells lining the renal tubule (red, E, G, H). DNA, DAPI (blue, F, G). Panel (H) is an enlargement of the boxed area in (E). Scale bars: 20 μm.

Considering that the *BicC* mutation causes renal cystogenesis in vertebrates [16,17,19,25,26], immunostaining was performed using species-specific antibodies to characterize expression and distribution of the BicC protein in wild-type Malpighian tubules and of its ortholog Bicc1 in kidney sections from C57BL/6 wild-type adult mice. *Drosophila* BicC was expressed in the Malpighian tubule epithelium (Fig 3A–3D). Similarly, mouse Bicc1 was found at highest levels in the renal tubule epithelium, with a much lower signal in the medullar region (Fig 3E–3H). Thus, consistent with the homologous primary structure [20], the *Drosophila* and the mouse BicC proteins are both expressed in the renal tubules, suggesting a possible functional conservation.

### *BicC* is necessary for proper Malpighian tubule function in *Drosophila*

**Fig 4:**
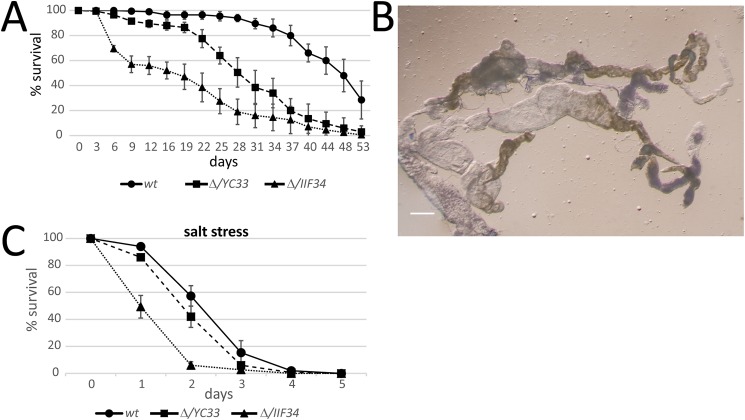
*BicC* flies exhibit severe renal defects and impaired survival. (A) Survival curves of fly populations (*n =* 200, standard deviations are shown) of wild-type (wt), *BicC*^*Δ/YC33*^ (Δ/*YC33*) and *BicC*^*Δ/IIF34*^ (Δ/*IIF34*). Compared to wild-type, both *BicC*^*Δ/YC33*^ and *BicC*^*Δ/IIF34*^ flies have impaired survival, reaching 50% respectively at 48, 28, and 16 days after adult eclosion. (B) Malpighian tubules dissected from a moribund *BicC*^*Δ/IIF34*^ fly showed severe deformities with large cysts containing apparent impacted materials; scale bar: 100 μm. (C) *BicC*^*Δ/YC33*^ and *BicC*^*Δ/IIF34*^ flies exhibited sensitivity to salt stress. Fly populations (wild-type, *BicC*^*Δ/YC33*^, *BicC*^*Δ/IIF34*^, *n =* 150, standard deviations are shown) placed in vials containing cornmeal agar with 0.5M NaCl were at a disadvantage and had shorter life span than the wild-type controls.

Cyst formation and interstitial fibrosis cause chronic kidney disease and kidney failure by age 55 in 50% of all PKD patients [8]. To investigate if the morphological defects in *BicC* mutant Malpighian tubules had phenotypic consequences, we assessed the viability of wild-type and mutant adult flies. Compared to wild-type, both *BicC*^*Δ/YC33*^ and *BicC*^*Δ/IIF34*^ mutant flies displayed impaired survival, with population decrease by day 9 to 91.5 ± 1.9% and 57 ± 6.6% survival for *BicC*^*Δ/YC33*^ and *BicC*^*Δ/IIF34*^ respectively, contrasted to 99.5 ± 1% survival for wild-type (*n* = 200; Fig 4A). Populations of *BicC*^*Δ/YC33*^ and *BicC*^*Δ/IIF34*^ mutants reached 50% survival at 28 and 16 days respectively, compared to 48 days for wild-type flies. Notably, *BicC*^*Δ/+*^ heterozygotes displayed similar survival to wild-type (S1 Fig) and were not further analyzed. Impaired viability correlated with renal degeneration. Malpighian tubules dissected from moribund flies were misshapen, displayed large fragile cysts, and often contained prominent impacted material (Fig 4B).

Malpighian tubule function was tested by placing flies in a cornmeal medium containing 0.5M NaCl. The hypertonic medium acts as a stress that may be used to reveal diminished Malpighian tubule function. Under the high-salt conditions survival of the wild-type was greatly impaired, with populations reaching 50% in ~2.2 days and 0% at 4–5 days. Compared to wild-type, *BicC*^*Δ/YC33*^ and *BicC*^*Δ/IIF34*^ fly lifespans were shortened, with populations steadily declining from exposure to high salt medium and reaching 50% survival at 1.8 and 1 days respectively (Fig 4C, *n* = 150). Differential survival did not appear to be due to starvation, because all genotypes appeared to feed normally on high salt food stained with food dye (S2 Fig). Taken together, our observations indicate that *BicC* is required for proper renal tubule function in *Drosophila*.

### BicC downregulates d-*myc* in the Malpighian tubule

**Fig 5:**
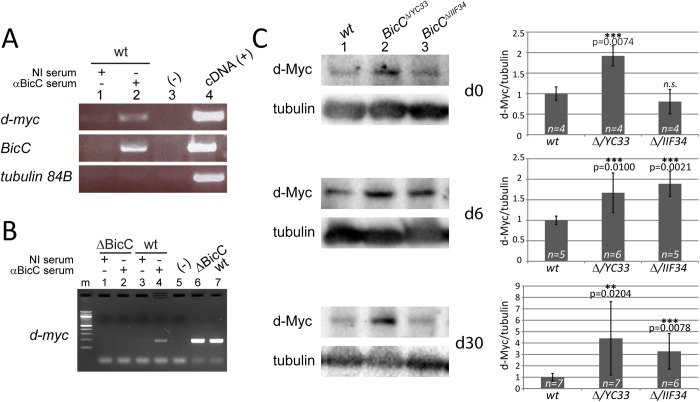
BicC regulates *d-myc* expression in the Malpighian tubule. (A) RT-PCR of RNA immunoprecipitated with either non-immune control serum (NI, lane 1) or BicC antiserum (αBicC, lane 2). A product specific for *d-myc* was amplified exclusively from the BicC immunoprecipitate, indicating that BicC regulates *d-myc* mRNA in the Malpighian tubule. Similar to the situation in the ovary [24], *BicC* primers used as positive control also yielded a specific product only in the immunoprecipitate. In contrast, *tubulin 84B* primers did not produce any amplification product, showing that the immunoprecipitation was specific (negative control). Lane 3: PCR negative control (-, no cDNA). Lane 4: PCR positive control (+, cDNA, Malpighian tubule total cDNA). (B) RT-PCR of control RNA immunoprecipitation from wild type and *Df(2L)RA5/Df(2L)Osp29* (*BicC* null, *ΔBicC*) Malpighian tubules. For both extracts RNP particles (RNPs) were captured with either non-immune control serum (NI, lane 1, 3) or BicC antiserum (αBicC, lane 2, 4). *d-myc*-specific amplification products were exclusively observed for wild type extracts with the RNP captured by the BicC antiserum, but not with those captured by the non-immune serum. Neither non-immune nor BicC antiserum recovered *d-myc* containing particles. RNAs extracted from small aliquots of the input extracts (respectively *ΔBicC* and wild-type) also produced distinct clear PCR products. (C) Representative immunoblots of extracts of Malpighian tubule (four pairs per lane) from wild-type (*wt*), *BicC*^*Δ/YC33*^ and *BicC*^*Δ/IIF34*^ flies probed for d-Myc and tubulin (left). Corresponding graphs of means ± standard deviations of d-Myc levels relative to tubulin (right). Values were calculated from independent biological replicas (*n*, indicated) and p values (Student’s t test) are shown (right). Myc/tubulin ratios were normalized to the wild-type average. d-Myc protein levels were generally higher in *BicC* mutants and increased with age.

In the *Drosophila* ovary BicC is a negative regulator of translation and functions by first binding to its mRNA targets [20,24]. To examine BicC role(s) in the Malpighian tubule, RNA-protein (RNP) complexes were immunoprecipitated from extracts of Malpighian tubules from wild-type flies with either the BicC antiserum or a non-immune serum. The content of *d-myc* RNA was specifically analyzed in the BicC-associated RNA pool, because of the important role c-Myc plays in kidney cystogenesis in mammals [38,39,40,41]. Unlike the non-immune control, an RT-PCR product for the *d-myc* mRNA was specifically obtained from BicC RNP immunoprecipitates (Fig 5A). In the ovary, BicC binds its own mRNA, downregulating its expression [24]. Using *BicC*-specific primers, we obtained an RT-PCR product from the BicC immunoprecipitates, suggesting that BicC could also bind its own mRNA in the Malpighian tubule, likely by an auto-regulatory loop as observed in the ovary. Conversely, *tubulin84B*-specific primers did not yield any RT-PCR products from either anti-BicC or control immunoprecipitates, indicating that the experimental conditions were selective for RNP complexes specifically containing BicC. Importantly, all primers amplified the expected size products when the reaction was spiked with cDNA synthesized from total RNA extracted from wild-type Malpighian tubules. To confirm the selective enrichment of the *d-myc* mRNA we repeated the RNP immunoprecipitation with immune and non-immune sera using extracts from flies either wild type or containing two overlapping deletions that remove the *BicC* gene, *Df(2L)RA5* and *Df(2L)Osp29 (ΔBicC)*. While a *d-myc*-specific PCR product was obtained from the wild type RNA associated with the BicC antiserum, no such product was observed for the RNPs recovered from the BicC deleted extract (Fig 5B). The immunoprecipitation with the non-immune serum yielded no amplification product from both extracts, suggesting that the BicC protein and the *d-myc* RNA may interact *in vivo*.

**Fig 6:**
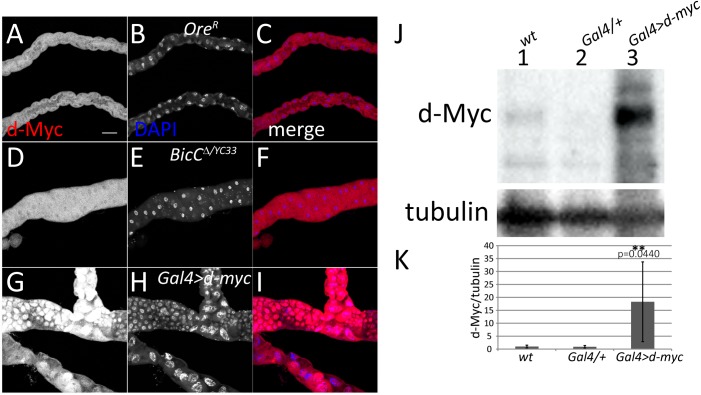
*d-Myc* overexpression in the Malpighian tubule causes severe cellular and tubular defects. Confocal sections of d-Myc immunostaining of Malpighian tubules from wild-type flies (A-C), *BicC*^*Δ/YC33*^ flies (D-F), and flies with d-Myc Gal4-driven over-expression in both principal and stellate cells (*Gal4>d-myc*, G-I). d-Myc (red), DNA (DAPI, blue). d-Myc over-expression in the Malpighian tubules caused severe defects. The irregular shape and density of nuclei are suggestive of tissue disorganization. All images in this panel were captured with identical settings and the signal for d-Myc staining in the *Gal4>d- myc* tubule (G) was saturated, indicating higher levels of d-Myc over-expression relative to those induced by *BicC* mutation. Scale bar: 50 μm. (J) Representative immunoblot of extracts of Malpighian tubules dissected from wild-type (*wt*) flies, flies heterozygote for the *Gal4-d-myc* construct (*Gal4/+*) and flies over-expressing *d-myc c724/+; c42/Gal4>d-myc* (*Gal4>d-myc*) probed for d-Myc and α-tubulin. (k) Graph of means ± standard deviations of d-Myc levels relative to tubulin from five independent biological replicas per each genotype. Values were normalized to the wild-type average; *p* values (Student’s t test) are shown. *d-myc* (d-Myc/tubulin) over-expression in Malpighian tubules ranged from three to over 30 times the levels in tubules of control flies.

In the ovary, BicC regulates negatively the expression of its own targets. Therefore, the levels of d-Myc protein in wild-type and *BicC* Malpighian tubules were compared relative to tubulin. Quantitative immunoblots of extracts from four pairs of Malpighian tubules which were dissected from flies of different ages (0, 6, 30 days old), revealed that tubules from BicC^*Δ/YC33*^ mutants contained from two to seven times more full-length d-Myc protein than wild-type tubules from flies of identical age that were raised in parallel (Fig 5C, *p* = 0.0074, 0.0100, 0.0204 respectively). d-Myc levels in *BicC*^*Δ/IIF34*^ tubules were also significantly above those of wild-type in the 6 and 30 days old samples (Fig 5C, *p* = 0.0021 and 0.0078 respectively).

c-Myc is upregulated in PKD kidneys [38,39,40,41,42,43] and its overexpression can induce renal cystogenesis in mice [39,40]. To characterize the consequences of d-Myc over-expression in the Malpighian tubules and to pinpoint how elevated d-Myc may contribute to the *BicC* cystic renal phenotypes, we overexpressed d-Myc both in principal and stellate cells (the main cell types of wild-type Malpighian tubules) of wild-type animals using the {*UAS-d-Myc*}^32^ [44] transgene and cell-specific Gal4 drivers *c42* and *c724* [45] to drive expression in principal and stellate cells respectively. Very few individuals over-expressing *d-myc* survived to adulthood. These rare escapers exhibited severely deformed Malpighian tubules, which were swollen and disorganized (compare Fig 6A–6C with D-F and G-I). Compared to *BicC*^*Δ/YC33*^, Malpighian tubules over-expressing d-Myc were severely deformed and, unlike the tubules of *BicC* mutant flies, did not appear to have extra tubular branching. d-Myc immunostaining (Fig 6A, 6D and 6G) and immunoblots of Malpighian tubule extracts from these flies (Fig 6J and 6K) confirmed that d-Myc was indeed substantially overexpressed relative to controls (3–34 X, *p* = 0.0440). Consistent with the observed severity of the *d-myc* over-expression defects, Myc levels in the Malpighian tubules of such flies were up to an order of magnitude higher relative to the *BicC* mutants.

**Fig 7:**
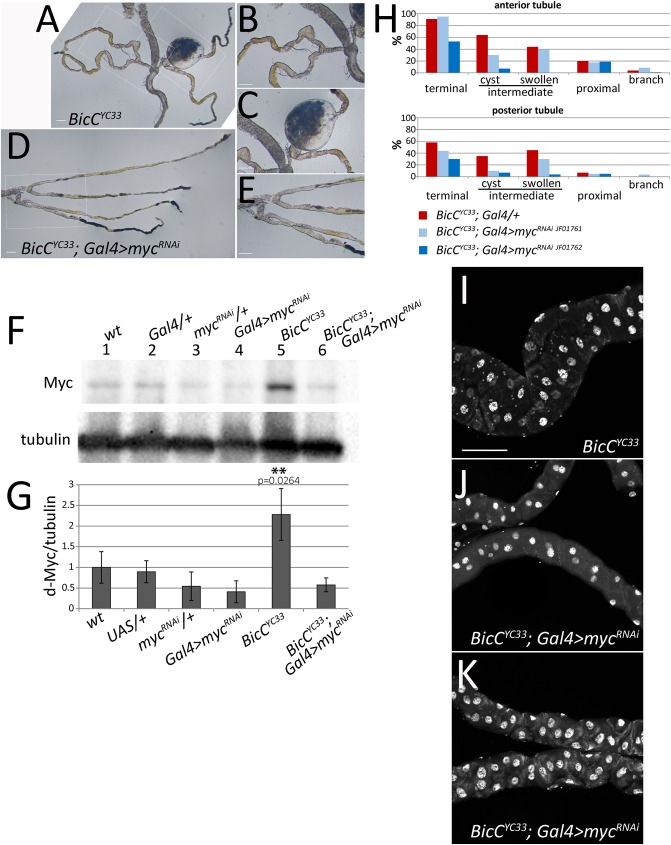
*d-myc* knockdown can rescue the Malpighian tubule defects of *BicC* mutants. Light microscopy of dissected Malpighian tubules from sibling *BicC*^*YC33/YC333*^ (A, B, C, control) and *BicC*^*YC33/YC33*^; *myc*^*RNAi*^ (D, E) flies in which RNAi was induced in both principal and stellate cells with the *c42* and *c724* drivers respectively. *d-myc* RNAi rescued greatly the morphology of the Malpighian tubule. Boxed regions in (A) and (D) are shown enlarged respectively in (B, C) and (E). (F) Representative d-Myc and tubulin immunoblots of extracts from five Malpighian tubules dissected from flies of the following genotypes: *wild-type* (*wt*); heterozygotes for the Gal4 driver *c724* and *c42* constructs (*Gal4/+*); heterozygotes for the d-*myc*^*RNAi*^ construct (*myc*^*RNAi*^/+); *myc* RNAi driven by *c724* and *c42* (*Gal4>myc*^*RNAi*^); *BicC*^*YC33/YC33*^ homozygotes (*BicC*^*YC33*^); *myc* RNAi driven in *BicC*^*YC33*^ homozygotes (*BicC*^*YC33*^; *Gal4>myc*^*RNAi*^). The latter two genotypes were sibling flies from the same crosses. (G) Corresponding graph summarizing quantitative immunoblots of means ± standard deviations of d-Myc/tubulin ratios from three independent biological replicas per each genotype. Values were normalized to the wild-type average. The *p* value (Student’s t test) is shown for the *BicC* mutants. Reducing *d-myc* expression in *BicC*^*YC33/YC33*^ mutants restored the d-Myc protein to control levels. (H) Cystic scoring of the *BicC*^*YC33/YC33*^*; Gal4/+* (red, *n = 100*) and *BicC*^*YC33/YC33*^*; Gal4/myc*^*RNAi*^ (blue) sibling flies. Results are shown for the lines TRiP.JF01762 (dark blue, *n =* 100) shown above, and TRiP.JF01761 (light blue, *n =* 80, see supplemental information). RNAi-induced d-Myc reduction decreased cystic deformities in the terminal and intermediate tubules. Results for the anterior and posterior tubules are shown separately. (I-K) Confocal sections of Malpighian tubules dissected from *BicC*^*YC33/YC33*^ flies (I, control) and *BicC*^*YC33/YC33*^; *myc*^*RNAi*^ flies (J, K) stained with DAPI. The distribution of cell nuclei in *BicC* Malpighian tubules (I) appeared disturbed (compared with wild-type in Figs 3B and 6B) with disrupted cell arrangement and cystic enlargements. Defects were largely rescued by reducing d-*myc* expression via RNAi specifically in the principal and stellate cells of the Malpighian tubules (J, K). Scale bar: 100 μm.

To verify if the Malpighian tubule cysts of the *BicC* flies were due at least in part to the observed up-regulation of d-Myc, we tested if cystogenesis could be suppressed by expression of a dsRNA transgene, *TRiP*.*JF01762*, [46,47] that targeted *d-myc* in both principal and stellate cells. The resulting flies (denoted as *BicC*^*YC33*^; *myc*^*RNAi*^) were dissected and Malpighian tubules from the various genotypes were examined by light microscopy to assess tubular morphology. Compared to the sibling *BicC*^*YC33/YC33*^ flies that displayed prominent cysts (Fig 7A–7C), *BicC*^*YC33/YC33*^; *myc*^*RNAi*^ flies exhibited substantial morphological rescue (Fig 7D and 7E).

Consistent with the results shown in Fig 5C, immunoblots of Malpighian tubule extracts from these genotypes revealed that, relative to tubulin, d-Myc levels in the *BicC*^*YC33/YC33*^ tubules were increased over two-fold compared to the wild-type (*p* = 0.0264). Compared to wild-type, *d-myc* RNAi slightly reduced d-Myc levels when induced in a wild-type background. In contrast, RNA interference in the *BicC*^*YC33/YC33*^; *myc*^*RNAi*^ flies reduced d-Myc to the levels of the wild-type and transgene heterozygote controls (Fig 7F and 7G). Myc RNAi in the *BicC*^*YC33/YC33*^ tubules reduced the incidence of cysts in the terminal and in the proximal tubule and could decrease swollen tubular sections, compared to Malpighian tubules from sibling *BicC*^*YC33/YC33*^ flies (Fig 7H). These results were confirmed with an independent line, TRiP.JF01761, which reduced Myc expression less efficiently than TRiP.JF01762 (S3 Fig). Staining with DAPI indicated that, in contrast to *BicC*^*YC33/YC33*^ tubules, which appeared enlarged with irregular distribution of principal cell nuclei, in the thinner-looking tubules from *BicC*^*YC33/YC33*^; *myc*^*RNAi*^ flies, cell nuclei appeared more regularly distributed and evenly shaped (Fig 7, compare I with J and K). We conclude that d-Myc up-regulation contributes substantially to the *BicC* cystic tubule phenotype.

### TOR pathway upregulation contributes to the cystic phenotype in *BicC* mutant flies

**Fig 8:**
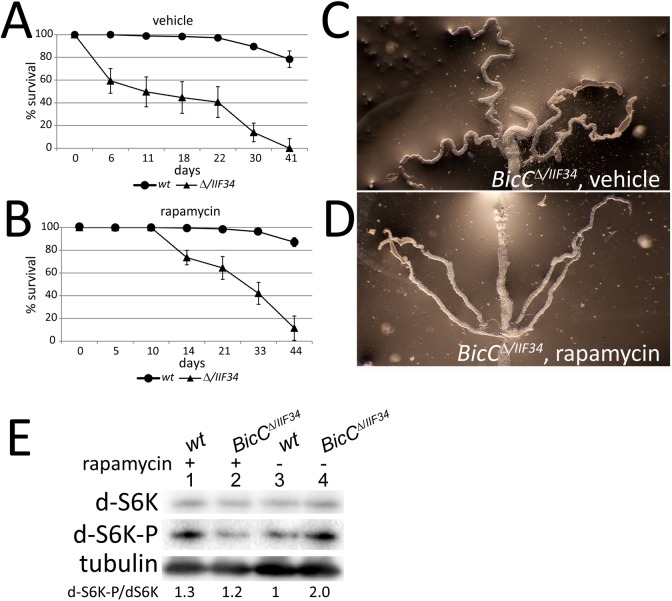
TOR upregulation and rapamycin rescue of *BicC* mutant flies. (A) Control wild-type (*wt*) and *BicC*^Δ*/IIF34*^ (Δ*/IIF34*) flies that were administered equal volumes of solvent (ethanol, vehicle) showed the characteristic impaired survival of *BicC* mutants compared to wild-type. (B) In contrast, sibling flies of both genotypes that were administered rapamycin exhibited markedly improved survival of the *BicC* mutants compared to control flies that were administered vehicle. A, B *n =* 200, standard deviations are shown. Rapamycin administration did not compromise survival of the wild-type flies. Rapamycin appeared to induce almost complete rescue at early time points and substantial rescue over time. Malpighian tubules of vehicle (C) and rapamycin-treated (D) *BicC*^*Δ/IIF34*^ flies showed marked morphological rescue with fewer cysts and more regular tubule structure. (E) Total and phosphorylated S6K immunoblots of extracts from wild type and *BicC*^*ΔIIF34*^ Malpighian tubules from flies administered rapamycin or vehicle for 8 days post-eclosion indicated apparent mitigation of the TOR up-regulation in the *BicC* mutants.

The activation of the mTOR pathway underlies many forms of renal cystogenesis, including both autosomal dominant and recessive PKD [6,7,48,49]. Since the observation that cystic progression was reduced in ADPKD patients undergoing post-transplantation immune suppressant therapy with rapamycin [7], the rapamycin derivatives Everolimus and Sirolimus have been examined in clinic and animal models to ameliorate kidney conditions and to delay cystic growth in the short term [50,51,52,53,54]. In light of the observed cystic defects in the *BicC* mutant flies, we tested if administration of rapamycin to adult flies could modify such cystic phenotype. The *BicC*^*Δ/IIF34*^ flies that were administered solvent alone (vehicle) exhibited the expected decreased fitness and rapid population decline relative to wild-type controls (50% survival at ~11 days; Fig 8A).

In contrast, *BicC*^*Δ /IIF34*^ mutants that were treated with 15 μM rapamycin exhibited substantial rescue, with no death observed before 10 days and 50% survival extended nearly two-fold to ~22 days (compare Fig 8A and 8B). Populations of *BicC* flies that were administered rapamycin eventually declined (Fig 8B), reminiscent of the short-term effectiveness of rapamycin treatment in rodent models of renal cystic disease [54]. Consistent with the observed rescue, the Malpighian tubules that were dissected from rapamycin-treated *BicC*^*Δ/IIF34*^ flies showed substantial improvements compared to those from vehicle-treated *BicC*^*Δ/IIF34*^ flies of the same age with rare, minimal-sized cysts and no extra branching, even at older ages (Fig 8C and 8D). Similar to observations of both *Drosophila* [55] and other treated organisms [56,57,58], rapamycin administration extended wild-type longevity.

Phosphorylation of the mTOR downstream target p70 ribosomal S6 kinase has been observed in both ADPKD human tissue and in mouse PKD models [7]. Comparative analysis of the *Drosophila* p70-S6 kinase [59] (hereby d-S6K) in the tubules from rapamycin or vehicle treated flies revealed that *BicC*^*Δ/IIF34*^ mutant tubules contained twice as much phosphorylated d-S6K relative to total d-S6K than wild-type (Fig 8E). In contrast, rapamycin-treated sibling flies, processed in identical conditions and in parallel, exhibited a similar ratio of phosphorylated vs. un-phosphorylated d-S6K (Fig 8E).

### An evolutionarily-conserved functional link between BicC, renal cystogenesis, and PKD

**Fig 9:**
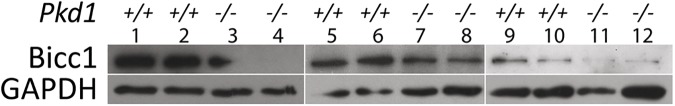
Bicc1 downregulation in *Pkd1*^*-/-*^ mice. (A) In accordance to our analyses of human microarrays of PKD patients, Bicc1 immunoblots of kidney extracts from newborn littermates *Pkd*^*+/+*^ and *Pkd1*^*-/-*^ showed that all *Pkd1*^*-/-*^ renal tissues exhibited considerable Bicc1 downregulation. GAPDH: loading control.

Two *BicC* point mutations were identified in patients with cystic kidneys [17]. To test if BicC was altered in the context of the molecular defects underlying PKD, we analyzed publicly available microarrays from *PKD1* ADPKD patients [60] and found that *BicC* was significantly downregulated in cystic relative to healthy renal tissue (*p* = 0.0196). This suggested that *BICC1* may be genetically downstream of *PKD1* in kidneys. Consistent with this possibility, we observed a severe decrease in Bicc1 protein levels in kidneys of *Pkd1*^*-/-*^ newborn mice compared to their *Pkd1*^*+/+*^ siblings (Fig 9, similar to reports of whole embryos in [61]).

## Discussion

In this work, we show that *BicC* mutation in *Drosophila* induced cellular and molecular defects typical of renal cystogenesis and PKD and that both human ADPKD tissue and *Pkd1*^*-/-*^ mice are characterized by decreased *BicC* function, which is likely to contribute to their characteristic cystic degeneration. *BicC* mutation in *Drosophila* caused progressive cystic degeneration of the renal tubule with remarkable similarity to the defects typical of human PKD. *BicC* mutant flies exhibited enlarged Malpighian tubules and fluid-filled cysts from the time of their hatching. Cyst number, size and severity increased over time. Moreover, the terminal and collector tubules appeared to be particularly affected, with frequent extra branching. Compared to wild-type flies, *BicC* mutants displayed reduced longevity and greater sensitivity to salt stress. Malpighian tubules dissected from moribund *BicC* flies were severely degenerated, indicative of compromised renal function.

We found that the BicC protein was similarly expressed in the cells of the renal tubular epithelium in both *Drosophila* and mice. BicC is an RNA binding protein that downregulates post-transcriptional expression of its target mRNAs in *Drosophila* [20,24]. Evidence for a link between BicC and renal cysts was obtained by immunopurification of BicC RNP particles from wild-type flies, which were found to contain specifically enriched *d-myc* mRNA, with up-regulation of Myc being a hallmark of cystic proliferation. Consistent with the profile of cysts, d-Myc protein was elevated in the Malpighian tubules of *BicC* flies. Moreover, d-Myc over-expression in the Malpighian tubules produced very deformed cystic tubules in rare survivors. The effects of d-Myc over-expression were more severe than *BicC* defects, likely attributable to the much higher d-Myc levels in the former (3-34X) than the latter (2-7X). Consistent with the possibility that the *d-myc* up-regulation in *BicC* mutants greatly contributes to the cystic phenotype, *d-myc* depletion via RNAi restored wild-type d-Myc protein levels in *BicC* tubules and substantially–albeit not completely–improved morphology. Therefore, de-regulation of other, still unknown, BicC targets is likely to contribute to the cystic degeneration of the renal tubule.

As in human and mice PKD tissue, *Drosophila* Malpighian tubules from *BicC* flies displayed elevated d-S6K phosphorylation, compared to wild-type, indicating TOR pathway activation. Of note, d-S6K phosphorylation may amplify the effects of *d-myc* over-expression [63]. The ratio between phosphorylated and un-phosphorylated d-S6K of BicC tubules was found to be twice as high as the wild-type, but rapamycin treatment restored wild-type levels. Consistently, rapamycin administration to the *BicC* flies markedly improved survival and reduced cystogenesis, albeit fly populations declined over the long term, as reported previously for PKD patients and rodent cystic models, indicating that repression of the TOR pathway rescued the cystic defects of the *BicC* Malpighian tubules. Rapamycin treatment was well-tolerated by wild-type flies, which displayed increased longevity. In vertebrate cystic models of PKD, long-term treatment with rapamycin has proven challenging because of habituation and possible toxicity [54,62]. Application of the *BicC* flies to elucidate the molecular mechanisms of cyst formation may thus empower the design of more effective therapeutic strategies.

Taken together, our findings are congruent with studies of vertebrate *BicC*: *BicC* function is needed for kidney homeostasis in vertebrates, mutations cause renal cysts in zebrafish, *Xenopus* and mice [16,19,26], and two *BicC* mutations were found in patients with infantile, unilateral renal cystic dysplasia [17]. Zebrafish BicC was also recently found in a protein complex with NPHP proteins [27] that are implicated in nephronophthisis [28]. This evidence suggests that evolutionarily conserved BicC functions are crucial for the physiology of the renal tubule and highlight the need for further study of the mechanism(s) and targets of BicC in the kidney and specifically in the renal tubule. Because decreased *BicC* expression was a feature of both microarrays from human *PKD1* tissue and *Pkd1*^*-/-*^ mouse kidney extracts, a strong nexus between the *BicC* and PKD phenotypes is beginning to emerge. BicC-mediated cellular functions such as post-transcriptional regulation of mRNAs may be part of evolutionarily-conserved pathways that are critical for kidney homeostasis, the malfunction of which leads to renal cystogenesis and PKD. The *Drosophila* Malpighian tubule promises to be a useful and genetically tractable system for further exploring the cellular and molecular changes that contribute to cystic kidney diseases and PKD.

## Methods

### Fly lines and survival assays

Flies were grown on cornmeal agar (Jazzmix) at room temperature (~23 degrees) and aged as indicated. For salt stress the cornmeal food was supplemented with crystalline NaCl to reach a final concentration of 0.5M NaCl. Food coloring was added to test feeding. Rapamycin (LC Laboratories) was dissolved in absolute ethanol and added to the fly food (final 15μM rapamycin, 1.5% ethanol). Unlike *Oregon*^*R*^ wild-type flies that were maintained by standard methods, *BicC* flies were generated by crossing CyO balanced stocks containing *Df(2L)RA5* (Bloomington Stock Center) and *BicC*^*YC33*^ or *BicC*^*IIF34*^ mutations[22] respectively and retrieving non-Cy progeny. The *BicC*^*YC33*^ and *BicC*^*IIF34*^ mutations gave rise to truncated proteins in the ovary, of which the former was present in low amounts (S4 Fig) and initially missed (see Fig 1 in [22]). The *BicC*^*IIF34*^ allele gave rise to the most severe phenotypes in both the Malpighian tubules and the ovary, suggesting it may behave as dominant negative. Because *Df(2L)RA5* double balanced flies are semi-lethal, the RNA interference transgenes and Gal4 drivers were crossed into *BicC*^*YC33*^ homozygotes. Note the *BicC*^*YC33*^ homozygotes were previously used in [24] to study ovarian development. *BicC* null flies were obtained by crossing *Df(2L)RA5* and *Df(2L)Osp29* (Bloomington Stock Center). Survival assays: for each genotype four cornmeal agar vials with 50 females and 12 males were set up. Flies were passed in fresh vials every three (normal and rapamycin-containing food) or one (high salt food) days and survivors counted. Percentage survival rates with standard deviations were plotted against time (Excel). For the rapamycin rescue, one day old flies (raised in Jazzmix) were placed in vials containing rapamycin or the same volume of solvent (ethanol, 1.5% final, vehicle control) and moved to fresh vials every three days. *d-myc* RNAi: the Gal4 drivers for the principal and stellate cells, *c42* and *c724* [45] and Gal4-inducible RNAi constructs targeting *d-myc*,: *y*^*1*^ *v*^*1*^*; p{y*^*+t7*.*7*^ *v*^*+t1*.*8*^ *= TRiP*.*JF01761}attP2* and *y*^*1*^ *v*^*1*^*; p{y*^*+t7*.*7*^ *v*^*+t1*.*8*^ *= TRiP*.*JF01762}attP2* (Bloomington *Drosophila* Stock Center) were introduced into the *BicC* background by crossing. To minimize possible environmental effects on gene expression, all the fly genotypes used in each assay were raised and processed simultaneously in parallel. *d-myc* overexpression: the UAS Gal4 transgene *w*^*1118*^*; P{w*^*+mC*^ *= UAS-Myc*.*Z}132* [44] (Bloomington *Drosophila* Stock Center) was induced via the *c724* and *c42* drivers at 18°C.

### Immunoblots

Malpighian tubules were micro-dissected in PBS. Four tubule pairs per each lane were lysed in RIPA, 1x Complete (Roche), 2X Laemmli sample buffer at 100°C, resolved on 10% SDS Laemmli polyacrylamide gels (PAGE) and transferred to PVDF.

Mice from *Pkd1* were backcrossed on C57BL/6J background for several generations and genotyped as in [64]. The protocols for *in vivo* mouse experiments were reviewed and approved by the Institut de recherches cliniques de Montreal—IRCM Animal Care Committee (ACC #2014–26), which follow the regulations and requirements of the Canadian Council on Animal Care (CACC). Dissected neonatal kidneys from *Pkd1*^*-/-*^ and control littermates were homogenized and lysed in RIPA buffer, 2mM PMSF, 2μg/ml aprotinin, and cleared by centrifugation. Aliquots of 25 μg were loaded in each well, resolved by 10% SDS-PAGE and transferred to nitrocellulose.

*Drosophila* immunoblots were probed with anti d-Myc antibody (rabbit, Santa Cruz Biotechnology, 1:2,000) or anti Thr398-phosphorylated p70 d-S6 Kinase (Cell Signaling, 1:1000), anti-d-S6K (1199, gift of G. Thomas) and anti α tubulin (mouse, 12G10, Developmental Studies Hybridoma Bank, 1: 50,000). Mouse immunoblots were probed with anti Bicc1 (Aviva Systems Biology) polyclonal and anti-GAPDH (Sigma) antibodies. Signal was revealed via horseradish peroxidase-conjugated secondary antibodies (Jackson Immunoresearch) and chemiluminescence on film (mouse) or recorded by ChemiDoc (BioRad) and analyzed (Image Lab 5.2, Excel, *Drosophila*). In this study the d-Myc positive band with electrophoretic mobility ~73 kDa (consistent with the expected d-Myc molecular weight) was quantified. Values were expressed as d-Myc/tubulin or d-S6K/tubulin intensity ratio, normalized to the average of the wild-type and plotted as means ± standard deviation. Where appropriate, *p* values were determined by Student’s t test. Similar trends were obtained utilizing actin for normalization. Antibody controls are shown in S5 Fig.

### Microscopy and immunofluorescence

Micro-dissected Malpighian tubules were photographed on a Leica MZ FLIII Fluorescence Stereomicroscope with Leica MZ series 10X/21B Widefield Adjustable eyepieces equipped with a Canon DS126201 EOS 5D MARK II, using visible light. Canon raw files were converted into TIF format (Adobe Lightroom 3.2). Images were merged and processed with Adobe Photoshop CS5. Immunofluorescence: dissected Malpighian tubules were fixed (20 min. in 4% paraformaldehyde 1X PBS 0.1% Tween-20), washed in PBT, incubated with primary antibody (anti-BicC [24] 1:1000, anti-d-Myc monoclonal 1:20, gift of Dr. Bruce Edgar) followed by Alexa Fluor 488 or 546 conjugated secondary antibodies (Molecular Probes) and DAPI, mounted (ProLong Gold, Molecular Probes), and imaged (Zeiss LSM710 confocal microscope). Acquired images were exported as TIF files (ZEN 2012), and processed with Adobe Photoshop CS5. 5μm thick section of paraffin-embedded, formaldehyde-fixed kidneys from adult mice were de-paraffinized, treated with NaBH_4_ for 30 min, washed with TBS, 1% SDS (5 min) and washed as described above prior to blocking (mouse IgG) and probing with 1:30 anti-Bicc1 polyclonal (Aviva Systems Biology), or blocking buffer (control), followed by Alexa-Fluor 546-conjugated secondary antibody and DAPI. Sections were mounted in ProLong Gold. Antibody controls are shown in S5 Fig.

### Immunoprecipitation and RNA assays

250 μl of packed, microdissected wild-type Malphighian tubules were lysed in ice-cold buffer (25 mM Hepes pH 6.8, 50 mM KCl, 1 mM MgCl_2_, 1 mM DTT, 125 mM sucrose, 100 U/ml RNasin (Promega) and cleared by centrifugation (10,000 g, 10 min, 4°C). The supernatant was brought to 0.2% Triton-X100, pre-cleared with protein A Sepharose, and incubated (2.5 hours, 4°C) with 25 μl of protein A Sepharose Fast Flow (GE Healthcare) pre-conjugated to either Bic-C anti-serum or non-immune control serum. The beads were washed in binding buffer, with 0, 250, 500, 600, mM NaCl and rinsed in 250 mM NaCl containing buffer, treated with proteinase K, extracted with phenol-chloroform and precipitated with ethanol. For the control RNA immunoprecipitation 150 Malpighian tubules from each genotype were lysed and processed as above. Extracts were divided equally and incubated with either non-immune or anti-BicC immune conjugates. RNA from the immunopurified RNPs was extracted with GENEzol (Geneaid) following recommended procedures. Recovered RNA was reverse transcribed with Superscript II and random primers (both Invitrogen) following the manufacturer’s recommendations. RT-PCR was performed with 1/10 of the reactions and the following gene specific primers: d-Myc (Forward, F: 5’CGATCGCAGACGACAGATAA3’, Reverse, R: 5’GGGCGGTATTAAATGGACCT3’), tubulin 84B (F: 5’TTACGTTTGTCAAGCCTCATAGC3’, R: 5’CTGAAGAAGGTGTTGAACGAGTC3’), BicC (F: 5’AATAGCTTTCCCGCACAACACAGC 3', R: 5' AAGGCAACTACGACCTATTGGCAC 3'), d-S6K (F: 5’ CAGTCAAGCATCCCTTCATAGT 3’, R: 5’ CGGTAGATGATGCCCAGTTT 3’), d-TOR (F: 5’ AGCTCTTTCGCTGTGCCAAT 3’, R: 5’ TCCAGTACGTTGTGGCTCGC 3’).

## Supporting information

S1 Fig Wild type and *BicC^Δ/+^* heterozygote flies have similar survival. Survival assays of populations of wild-type (*wt*) and *BicC* heterozygotes for the *BicC* deletion (*Δ/+*, *n* = 200, with standard deviations) showing that *BicC* hemizygotes and wild-type flies displayed similar survival.
(TIF)

S2 Fig Flies were fed on high salt vials containing green food dye for 30 hours, frozen and mounted on modeling clay for photography. For image clarity legs were clipped.
(TIF)

S3 Fig (A) Representative d-Myc and tubulin immunoblots of extracts from five Malpighian tubules dissected from flies of the following genotypes: *d-myc* RNAi driven in *BicC*^*YC33/YC33*^ homozygotes (*BicC*^*YC33*^; *Gal4>myc*^*RNAi*^); *BicC*^*YC33/YC33*^ homozygotes (*BicC*^*YC33*^, sibling to the previous flies); *wild-type* (*wt*); heterozygotes for *BicC*^*YC33*^ and the *c42* Gal4 driver. The TRiP line used in these assays was *JF01761*. (B) Corresponding graph summarizing quantitative immunoblots of means ± standard deviations of d-Myc/tubulin ratios from independent biological replicas per each genotype (*n*, indicated). Values were normalized to the wild-type average; *p* values (Student’s t test) are shown for the *BicC* mutants and rescued flies. For the latter, significance was computed compared to wild type (top) and *BicC* mutant (below). Reducing *d-myc* expression in *BicC*^*YC33/YC33*^ mutants decreased the d-Myc protein.
(TIF)

S4 Fig *BicC* alleles *BicC^YC33^* and *BicC^IIF34^* produce truncated proteins. BicC immunoblots of extracts from dissected ovarian stages 1–9 (20 μg/lane) of the following genotypes: *Ore*^*R*^ (wild type, wt, lane 1); *BicC*^*Δ/YC33*^ (lane 2); *BicC*^*Δ/IIF34*^ (lane 3); *w EGFPnos* (E, Forrest *et al*. 2004, lane 4); *w EGFPnos; BicC*^*Δ/YC33*^ (E; *BicC*^*Δ/YC33*^; lane 5); *w EGFPnos; BicC*^*Δ/IIF34*^ (E; *BicC*^*Δ/IIF34*^; lane 6) show that both *BicC*^*Δ/YC33*^ and *BicC*^*Δ/IIF34*^ flies produced truncated BicC proteins (asterisks), compared to the full-length BicC protein found in wild type and the *w EGFPnos* ovaries (lanes 1 and 4, respectively). The smudge at ~ 100 kDa in lane 6 was due to spill over from the sample in the next well.
(TIF)

S5 Fig Antibody controls. (A) Whole d-Myc immunoblot for the gel in Fig 7F. d-Myc is indicated with an asterisk. The molecular size marker is shown (colorimetric image, left) and corresponding sizes are specified. Red indicates areas of over-exposure. (B) Immunoblot of Malpighian tubule extracts from *myc*^*P0/dm1*^, *Ore*^*R*^ (wt), *Gal4>TRiP*.*JF01761*, *Gal4>TRiP*.*JF01762*. (A, B): rabbit polyclonal anti-d-Myc. (C) Confocal section of Myc immunostaining of *Ore*^*R*^ (wt) with anti-Myc monoclonal B10. (D) Confocal section of Myc immunostaining of *myc*^*P0/dm1*^ Malpighian tubules with anti-d-Myc monoclonal B10; *myc*^*P0/dm1*^ was obtained by crossing strong hypomorphic mutants [44,65]. (E) Epifluorescence microscopy of a 5 μm kidney section from C57BL/6 mice shows Bicc1 accumulation in the cells lining the renal tubule and DNA (DAPI, blue). This is the same panel shown in (Fig 3E–3G). (F) A 5 μm kidney section from C57BL/6 mice was processed in parallel and identical conditions to (E), except for the addition of the primary anti-Bicc1 antibody. All image pairs were captured in identical conditions and the corresponding samples processed in parallel. (G) Epifluorescence microscopy of a mosaic Malpighian tubule displaying a single cell expressing a long dsRNA targeting BicC (*Valium 20 P{TRiP*.*HMS01407}*) and marked by GFP co-expression, surrounded by neighbouring wild-type cells. The clone boundaries are indicated. (C-G): DNA (DAPI), blue. Scale bars indicated.
(TIF)

S1 Text Supporting methods describing the *d-myc* alleles in S5 Fig and procedures for making protein extracts and immunoblots from dissected ovaries as displayed in S4 Fig with associated references. (DOCX)
