# Supplementary figures and images for: Bicaudal C mutation causes myc and TOR pathway up-regulation and polycystic kidney disease-like phenotypes in Drosophila
Source: PLoS Genet. 2017 Apr 13;13(4):e1006694. doi: 10.1371/journal.pgen.1006694 (PMC5390980; doi:10.1371/journal.pgen.1006694)

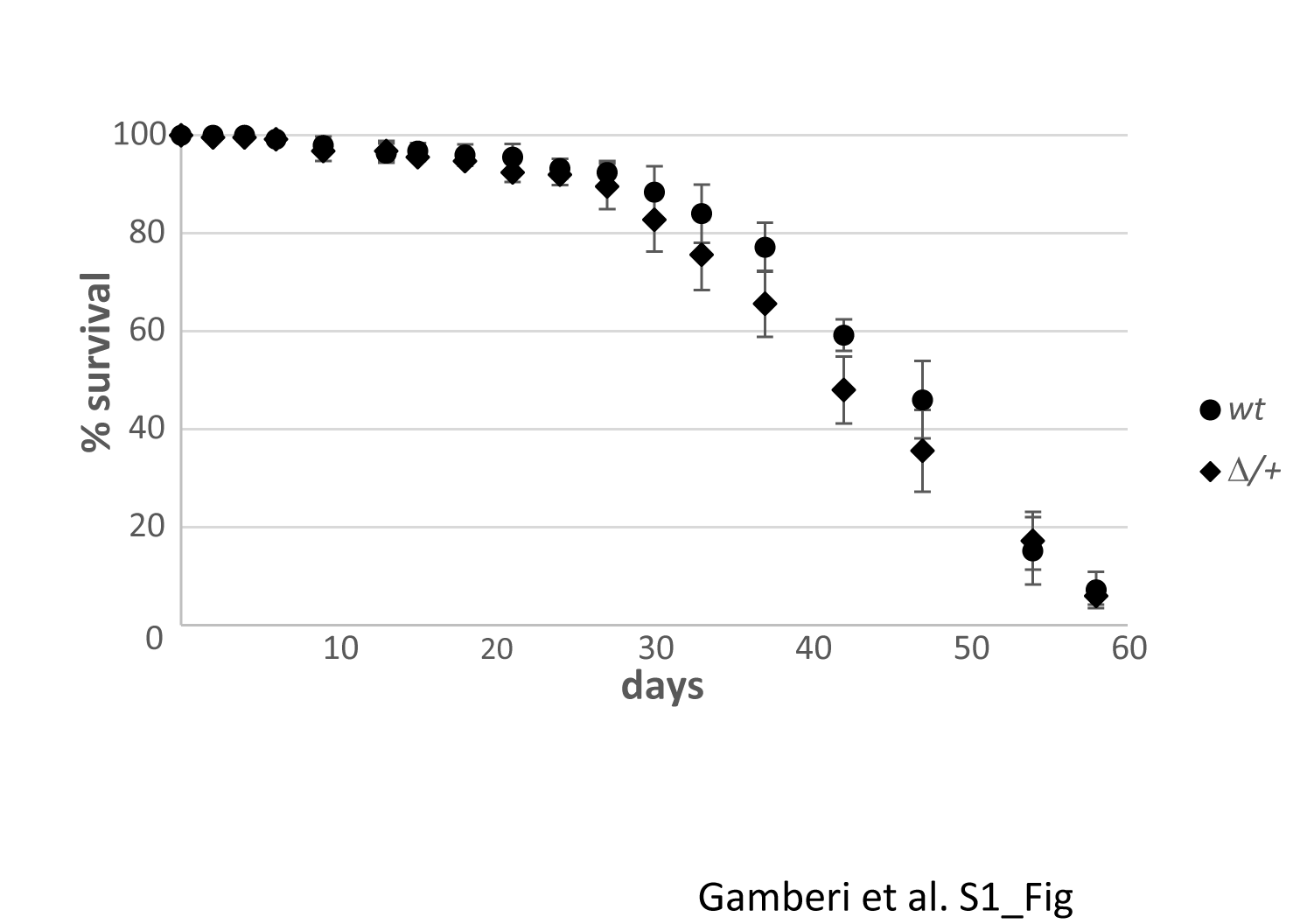

Supplement: S1 Fig — Survival assays of populations of wild-type (wt) and BicC heterozygotes for the BicC deletion (Δ/+, n = 200, with standard deviations) showing that BicC hemizygotes and wild-type flies displayed similar survival. (TIF) [file pgen.1006694.s001.tif]

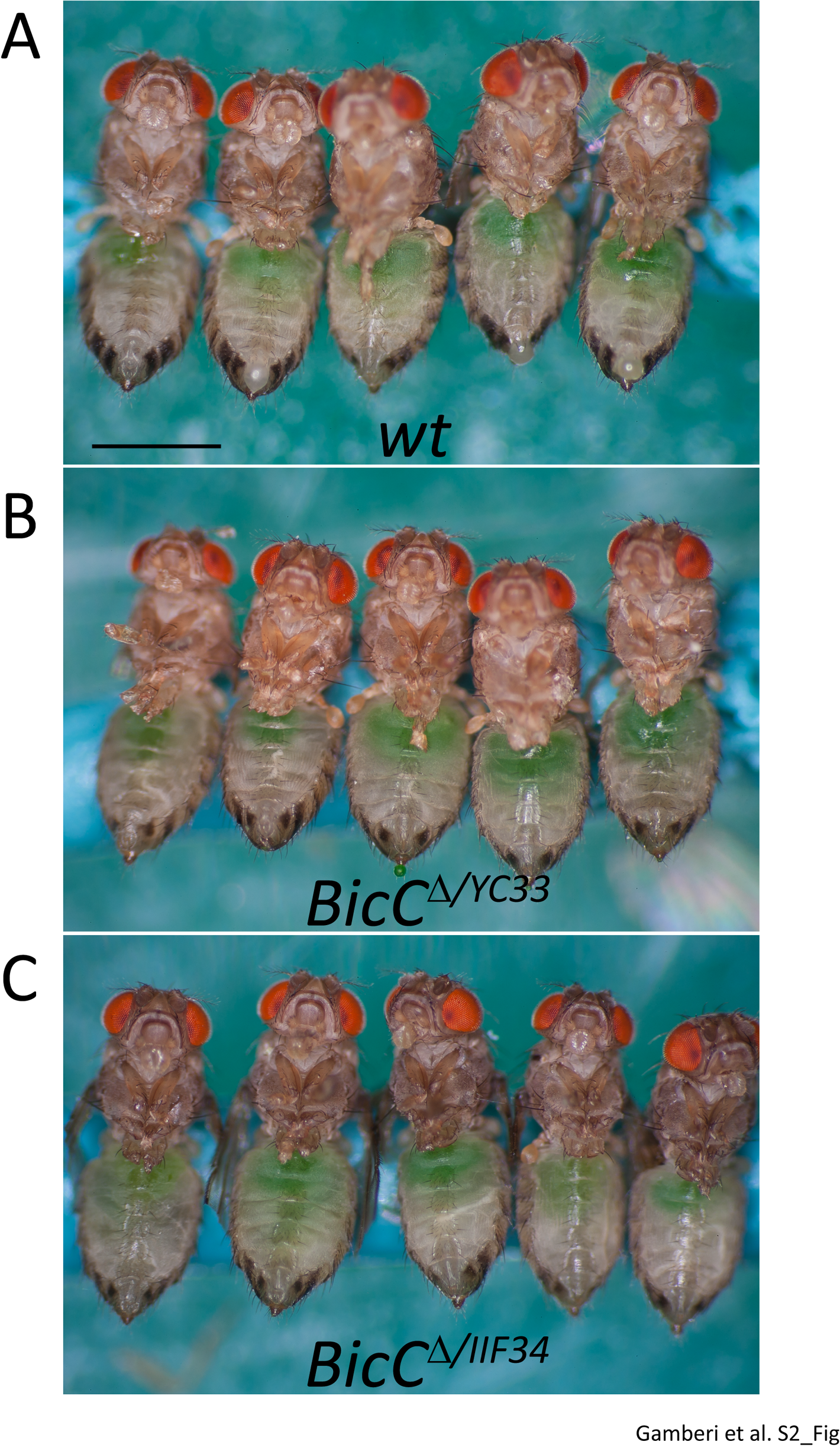

Supplement: S2 Fig — For image clarity legs were clipped. (TIF) [file pgen.1006694.s002.tif]

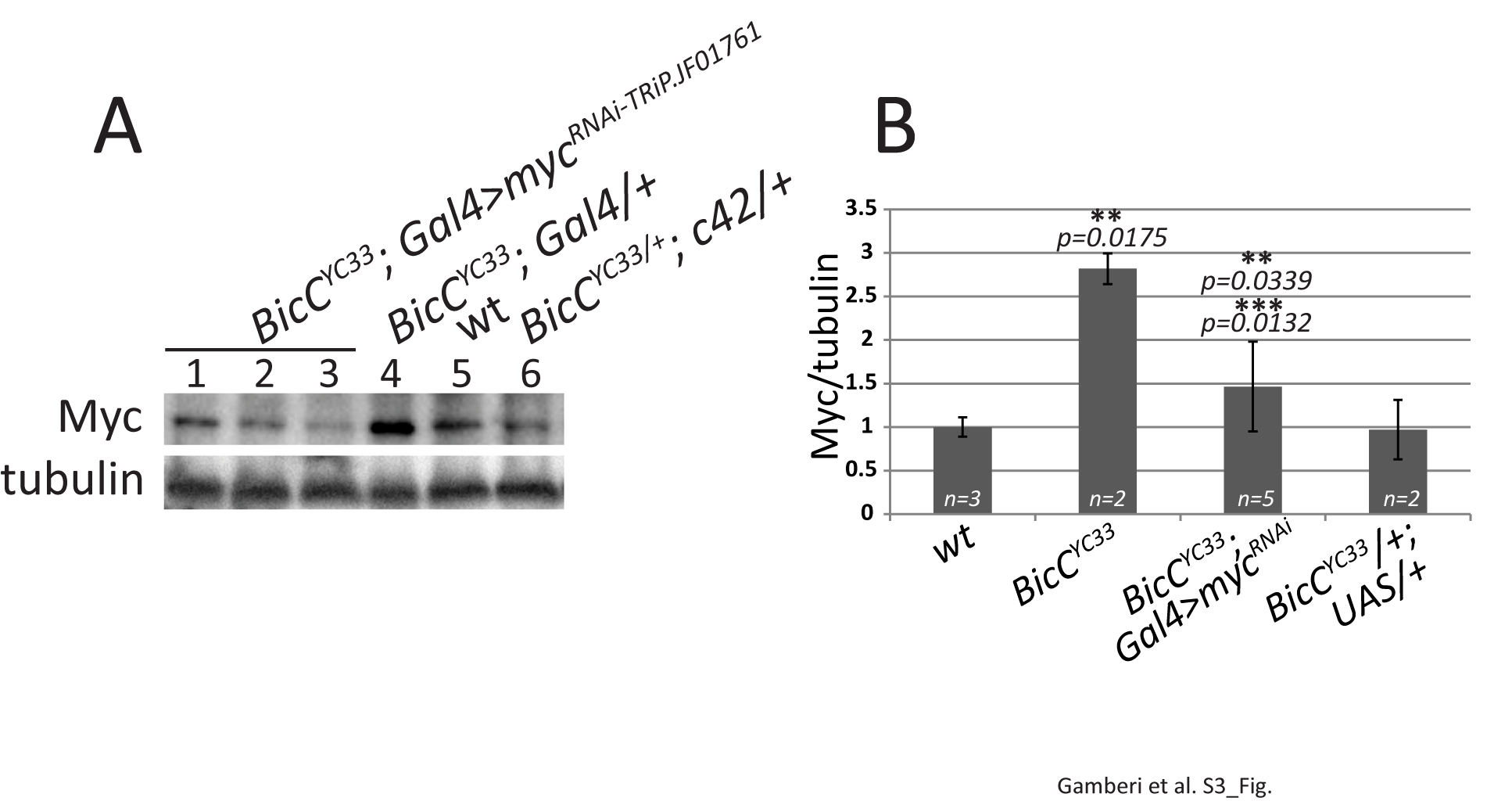

Supplement: S3 Fig — (A) Representative d-Myc and tubulin immunoblots of extracts from five Malpighian tubules dissected from flies of the following genotypes: d-myc RNAi driven in BicCYC33/YC33 homozygotes (BicCYC33; Gal4>mycRNAi); BicCYC33/YC33 homozygotes (BicCYC33, sibling to the previous flies); wild-type (wt); heterozygotes for BicCYC33 and the c42 Gal4 driver. The TRiP line used in these assays was JF01761. (B) Corresponding graph summarizing quantitative immunoblots of means ± standard deviations of d-Myc/tubulin ratios from independent biological replicas per each genotype (n, indicated). Values were normalized to the wild-type average; p values (Student’s t test) are shown for the BicC mutants and rescued flies. For the latter, significance was computed compared to wild type (top) and BicC mutant (below). Reducing d-myc expression in BicCYC33/YC33 mutants decreased the d-Myc protein. (TIF) [file pgen.1006694.s003.tif]

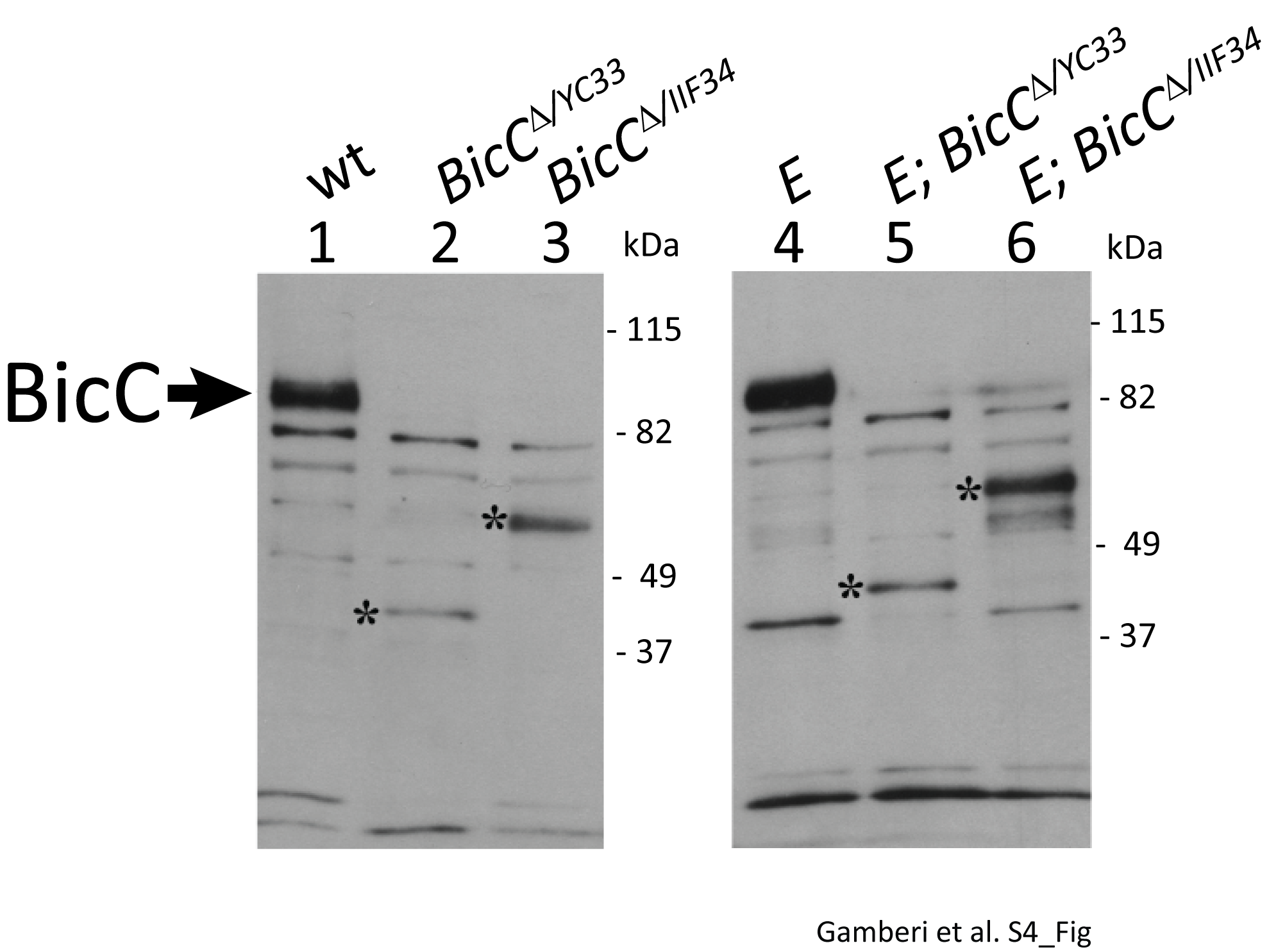

Supplement: S4 Fig — BicC immunoblots of extracts from dissected ovarian stages 1–9 (20 μg/lane) of the following genotypes: OreR (wild type, wt, lane 1); BicCΔ/YC33 (lane 2); BicCΔ/IIF34 (lane 3); w EGFPnos (E, Forrest et al. 2004, lane 4); w EGFPnos; BicCΔ/YC33 (E; BicCΔ/YC33; lane 5); w EGFPnos; BicCΔ/IIF34 (E; BicCΔ/IIF34; lane 6) show that both BicCΔ/YC33 and BicCΔ/IIF34 flies produced truncated BicC proteins (asterisks), compared to the full-length BicC protein found in wild type and the w EGFPnos ovaries (lanes 1 and 4, respectively). The smudge at ~ 100 kDa in lane 6 was due to spill over from the sample in the next well. (TIF) [file pgen.1006694.s004.tif]

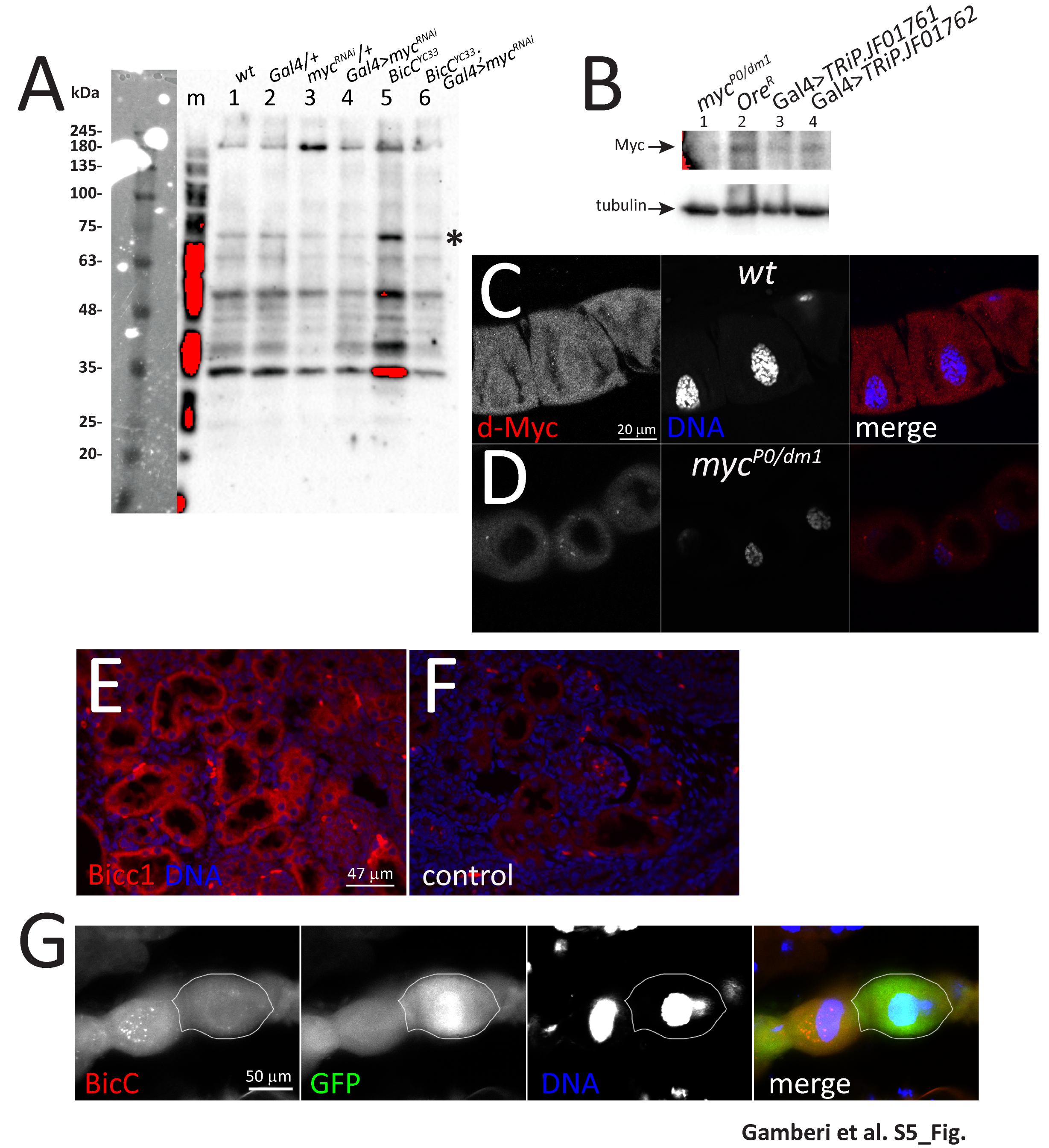

Supplement: S5 Fig — (A) Whole d-Myc immunoblot for the gel in Fig 7F. d-Myc is indicated with an asterisk. The molecular size marker is shown (colorimetric image, left) and corresponding sizes are specified. Red indicates areas of over-exposure. (B) Immunoblot of Malpighian tubule extracts from mycP0/dm1, OreR (wt), Gal4>TRiP.JF01761, Gal4>TRiP.JF01762. (A, B): rabbit polyclonal anti-d-Myc. (C) Confocal section of Myc immunostaining of OreR (wt) with anti-Myc monoclonal B10. (D) Confocal section of Myc immunostaining of mycP0/dm1 Malpighian tubules with anti-d-Myc monoclonal B10; mycP0/dm1 was obtained by crossing strong hypomorphic mutants [44,65]. (E) Epifluorescence microscopy of a 5 μm kidney section from C57BL/6 mice shows Bicc1 accumulation in the cells lining the renal tubule and DNA (DAPI, blue). This is the same panel shown in (Fig 3E–3G). (F) A 5 μm kidney section from C57BL/6 mice was processed in parallel and identical conditions to (E), except for the addition of the primary anti-Bicc1 antibody. All image pairs were captured in identical conditions and the corresponding samples processed in parallel. (G) Epifluorescence microscopy of a mosaic Malpighian tubule displaying a single cell expressing a long dsRNA targeting BicC (Valium 20 P{TRiP.HMS01407}) and marked by GFP co-expression, surrounded by neighbouring wild-type cells. The clone boundaries are indicated. (C-G): DNA (DAPI), blue. Scale bars indicated. (TIF) [file pgen.1006694.s005.tif]
