# Supplementary material for: Bicaudal C mutation causes myc and TOR pathway up-regulation and polycystic kidney disease-like phenotypes in Drosophila
Source: PLoS Genet. 2017 Apr 13;13(4):e1006694. doi: 10.1371/journal.pgen.1006694 (PMC5390980; doi:10.1371/journal.pgen.1006694)
Supplement: S1 Text — (DOCX) [file pgen.1006694.s006.docx]

**Supporting Information**

**Supporting Methods**

*Myc^dm1^* and *myc^P0^* (Johnston et al., 1999, Gallant et al., 1996) were, respectively, from the Bloomington Stock Center, and a gift of D. Grifoni. For S4 Fig manually dissected ovarian stages 1-9 were homogenized in 30 mM Hepes pH 7.2 300 mM KCl 0.5 mM EDTA 10% glycerol 1 mM DTT 4X Complete™ (Roche) 2 mM PMSF and centrifuged at 10,000 rpm and 4 °C. Supernatants were quantified by Bradford assay using reference bovine serum albumin (Pierce). Extracts were resolved on 6-12% SDS-PAGE Laemmli gels, transferred on Hybond-C extra (Amersham), probed with 1:1000 BicC antiserum (Chicoine *et al*. 2007) as described in the Methods section and followed by chemiluminescence detection (GE Healthcare).

**Supporting References**

1. Chicoine J, Benoit P, Gamberi C, Paliouras M, Simonelig M, et al. (2007) Bicaudal-C recruits CCR4-NOT deadenylase to target mRNAs and regulates oogenesis, cytoskeletal organization, and its own expression. Developmental cell 13: 691-704.

2. Forrest KM, Clark IE, Jain RA, Gavis ER (2004) Temporal complexity within a translational control element in the nanos mRNA. Development 131: 5849-5857.

3. Gallant P, Shiio Y, Cheng PF, Parkhurst SM, Eisenman RN (1996) Myc and Max homologs in Drosophila. Science 274: 1523-1527.

4. Johnston LA, Prober DA, Edgar BA, Eisenman RN, Gallant P (1999) Drosophila myc regulates cellular growth during development. Cell 98: 779-790.
